# L-SHADE optimized learning framework for sEMG hand gesture recognition

**DOI:** 10.1038/s41598-025-20076-9

**Published:** 2025-10-21

**Authors:** Naveen Gehlot, Ankit Vijayvargiya, Ashutosh Jena, Rajesh Kumar, Surender Hans, Priyanka Harjule

**Affiliations:** 1 Present Address: Department of Instrumentation and Control Engineering, Manipal Institute of Technology, Manipal Academy of Higher Education, Manipal, India; 2 Department of Electrical Engineering, Malaviya National Institute of Technology, 302017 Jaipur, Rajasthan India; 3 Dhirubhai Ambani Institute of Information and Communication Technology, Gandhinagar, India; 4 Department of Human Anatomy and Physiology, Faculty of Health Sciences, University of Johannesburg, 2094 Johannesburg, South Africa; 5 Department of Mathematics, Malaviya National Institute of Technology, 302017 Jaipur, Rajasthan India

**Keywords:** Electromyography signal, Optimization techniques, Machine learning models, Human machine interaction, Hand gesture recognition, Hyperparameter, Biotechnology, Health care, Engineering, Mathematics and computing

## Abstract

In recent years, Hand Gesture Recognition (HGR) devices have been designed to recognize gestures in real time using machine-learning classifiers (MLCs). However, the performance of these classifiers heavily relies on the tuning of their hyperparameters on real-time data. In this regard, this study provides a Linear Population Size Reduction Success-History Adaptation Differential Evolution (L-SHADE)-based optimized Extra Tree (ET) MLC framework for HGR. The study includes real-time sEMG signals from two forearm muscles to capture six distinct hand gesture movements. To recognize the gesture, this work employed ten MLCs. Among these ET classifier demonstrates the highest accuracy without optimizing the hyperparameters. To further enhance performance, ten optimization algorithms, along with the ET classifier, are considered, where the L-SHADE optimized ET framework outperforms the others. To validate the proposed framework, a consistent system environment has been used for both acquired and public datasets. On the acquired data, the mean accuracy improves from 84.14% to 87.89% using ET with the L-SHADE optimization framework while the mean computational time is reduced from 8.62 to 3.16 milliseconds. Similarly, the publicly available 15-hand gesture classification dataset demonstrated a mean accuracy improvement of more than 3.0%.

## Introduction

The hand plays a vital role in human interaction with the external environment. A hand can convey the emotions of a human through different hand gestures and can sensitively perceive changes in the environment. The hand affects the daily life activities of amputees. Amputations mainly result from pathology, injuries, and accidents^1,2^. As per the World Health Organisation (WHO), estimates that approximately 16% of the global population, or 1.3 billion people, live with a significant disability^3^. Of these, arm amputation affects about 3 million people globally and accounts for almost 2.4 million cases recorded in developing countries. In recent years, engineering applications in the biomedical and robotic fields have become more prevalent, mainly in medicine, remote surgery, assistive technologies, pacemakers^4^, etc. Due to the growing population and demand, and for some specific applications like assistance to physically impaired people or remote surgery, the assistive tools need to be highly precise and accurate. For instance, writing on paper with a prosthetic limb or performing remote surgery requires greater flexibility and accuracy. Therefore, a robotic hand is among the most effective solutions for armless and handless people^5^. To create an autonomous system for amputees, precise control of the robotic hand using signals from the human body or consciousness is required. Researchers have investigated the use of electromyogram (EMG) and electroencephalogram (EEG) signals in this scenario^6^. The EEG records the brain’s electrical activity and is captured by placing electrodes directly in contact with the human head. The EMG, on the other hand, records electrical signals generated by skeletal muscles^7^.

EMG is the signal utilised most frequently in prosthetic applications^8^. Using EMG to differentiate between hand gestures is more straightforward than other signals due to the physiological processes underlying its development in the skeletal muscles. As a result, biological applications and clinical diagnostics are the primary motivations for using EMG signals. There are two methods for collecting the EMG signals from muscles: invasive and non-invasive. In invasive methods, needles or wires are injected into the muscles to collect the muscle action potential, whereas, in the case of non-invasive methods, electrodes are pasted on the skin surface, from where the field generated by muscle fiber potential is collected^9^. The signal collected from the invasive method is called intramuscular EMG (iEMG), and the non-invasive method is called the surface EMG (sEMG) signal. The sEMG signal is frequently employed because iEMG may sometimes rupture tissue and cause discomfort^10,11^.

Adopting assistive technology based on sEMG signals can improve the quality of an amputee’s day-to-day life. Pradhan et al.^12^ have presented the use of INA128 to collect sEMG data for capturing hand gestures using Texas ADS1294, which is specifically designed for biomedical applications. Pancholi et al.^13^ utilized the ADS1298 IC, similar to the ADS1294, for biomedical signal acquisition. Ankit et al.^14^ considered the Myoware sensors to collect sEMG data for the activity classifications. Even though there are several signal conditioning tools and high-level EMG signal collection devices on the market, such as Biopac and BioNomadix, they are known for making high-quality, research-grade EMG devices and have a long-standing reputation for accurate signal capture and reliable data^15^.

The EMG signals collected through these acquisition devices for different activities, such as hand gestures, gait activities, etc., can be used for real-time applications such as robotics and prosthesis control^16^. Ulkir et al.^17^ demonstrated a fuzzy logic-based classification of acquired muscular signals, which is easy while dealing with three handcrafted features (Root mean square, Wavelength, Kurtosis) to classify signals. However, new fuzzification rules must be prepared as the number of features increases, and the task becomes complicated. To overcome these, machine learning is introduced as a solution. Maria et al.^18^ have collected the data with the help of four-channel acquisition devices for six different hand gesture activities. They extracted six time and frequency domain features, such as Willison Amplitude (WAMP), Variance (VAR), Mean Absolute Value (MAV), Waveform Length (WL), Median Frequency (MDF), Mean Frequency (MNF), and then applied three machine learning models for the classification of different gestures. Kuzborskij et al.^19^ consider the publicly available sEMG signals of 52 different gestures performed by twenty-seven participants and extracted seven time and frequency domain features. The gestures are then classified and identified using four distinct machine learning classifiers, namely Linear Discriminant Analysis (LDA), Multilayer Perceptron (MLP), k-Nearest Neighbour (KNN), and Support Vector Machine (SVM). Omari et al.^20^ extracted ten handcrafted features and analyzed the impact of the combinations of features with four machine learning models to evaluate the best combinations of features with the highest classification rate for gesture recognition. Song et al.^21^ used a wearable smart sEMG recorder integrated with gradient boosting to recognize the hand gesture.

According to the literature, machine learning models utilize hyperparameters, which have preset parameters. These parameters may be configured during the training phase of the model as well as the initial configuration phase. Due to the default value of these models, it does not guarantee the maximum level of efficacy^22^. As a result, machine learning models with an extensive range of hyperparameter values can be considered to construct the best-performing machine learning model. Determining the optimal hyperparameter value for a machine learning model is often referred to as hyperparameter tuning^23^. Sajjad Nematzadeh et al.^24^ have utilized Genetic Algorithm (GA) and Grey Wolf Optimization (GWO) based hyperparameter tuning of various machine learning algorithms. In this classification, diverse types of biological, biomedical, and natural data sets are classified, including molecular interactions, clinical diagnosis, cancer, RGB images of human skin, behavior-related predictions, and X-rays of COVID-19 and cardiomegaly patients. B. Chitra and colleagues^25^ use a densenet121 deep learning model with hyperparameter optimization using Atom Search Optimization (ASO) to detect cervix cancer. Marius Geitle et al.^26^ have conducted a comparative analysis of three optimization techniques (L-SHADE, random search, and adaptive random search) with an XGboost machine learning model to modify the hyperparameter and found that L-SHADE outperforms the others for different publicly available datasets. K. Jayaprakash et al.^27^ have implemented the Artificial Rabbits Optimization (ARO) with deep learning models for plant disease classification. Woo-Young Lee et al.^28^ have used the Harmony Search (HS) to tune the hyperparameters of Convolutional Neural Network (CNN) for image classification. Nazrul Islam et al.^29^ have presented the water quality prediction based on Elman Neural Network (ENN) and tuned the hyperparameters using Artificial Ecosystem Optimization (AEO). Ismail Damilola Raji et al.^30^ have applied Stochastic Gradient Descent (SGD), Bayesian Optimization (BO), GA, Particle Swarm Optimization (PSO), and Biogeography-Based Optimization (BBO) to tune the hyperparameters of different machine learning models for the benchmark UCI and MNIST datasets. Xiuwu Sui et al.^31^ have classified the six upper limb activities using PSO with improved SVM. The penalty parameter and kernel function parameters of SVM are optimized using the PSO. Similarly, Siqiao Yang et al.^32^ considered a GA for tuning the parameters of SVM for the classification of nine different hand activities.

Recent developments in biomedical signal processing have shown the effectiveness of hybrid techniques combining multiple analytical approaches. For biomedical signal processing, Eraslan et al.^33^ successfully used hybrid adaptive neuro-fuzzy inference systems for dynamic time warping-based authentication, while Ozturk et al.^34^ investigated unified frameworks integrating t-SNE and dynamic time warping for biomedical signal-based biometric applications, offering insightful information for optimizing signal processing frameworks. Also, in parallel, recent advancements in deep learning (DL) approaches have gained significant attention for their ability to extract complex features from biomedical signals and classify gestures. Recent studies^35–39^ explore human-machine interaction and intuitive control in various applications such as gesture recognition, breathing pattern recognition, and gait analysis using DL-based techniques. Moreover, compared to traditional machine learning methods, these recent deep learning models often require large datasets and high-performance computational units which making the systems costlier and bulkier, which may hinder their use in daily-life activities. These recent advancements in biomedical signal processing and machine learning optimization highlight the need for hyperparameter tuning techniques in gesture recognition systems, which aligns with the objectives of this study. In the applications of the biomedical field, a slight change in accuracy is of the utmost value. The precise recognition of hand gestures is also essential because it has numerous applications, such as robotics and prosthesis control. As per the literature survey, there is relatively less work on the tuning of hyperparameters of machine learning models employing an optimization method for hand gesture recognition. Along with the recent growth of research in sEMG-based HGR, relatively few studies have explored the comparative analysis of the advanced metaheuristic techniques for hyperparameter optimization. This study provides a Linear Population Size Reduction Success-History Adaptation Differential Evolution (L-SHADE)-based optimized Extra Tree (ET) MLC framework for HGR. This study also provides a systematic review of ten machine learning models used to recognize six different hand gestures, and then ten distinct optimization techniques are applied to tune the hyperparameters of the machine learning algorithm, which yields the best results.

The main contributions that have been presented in this research article are as follows:

1. sEMG signal-based recognition of hand gestures using an optimized extra tree framework by tuning the hyperparameters using L-SHADE optimization.
2. L-SHADE optimized extra tree framework is compared with ten optimization approaches for the hyperparameter tuning.
3. Comparative analysis of ten machine learning models are applied for the classification of hand gestures.
4. sEMG signal of six different hand gestures is acquired from the two forearm muscles of four participants (two males and two females) using BIOPAC MP150.
5. Performance parameters of machine learning models without hyperparameter tuning are also examined and compared to the proposed tuned machine learning models.

**Fig. 1:**
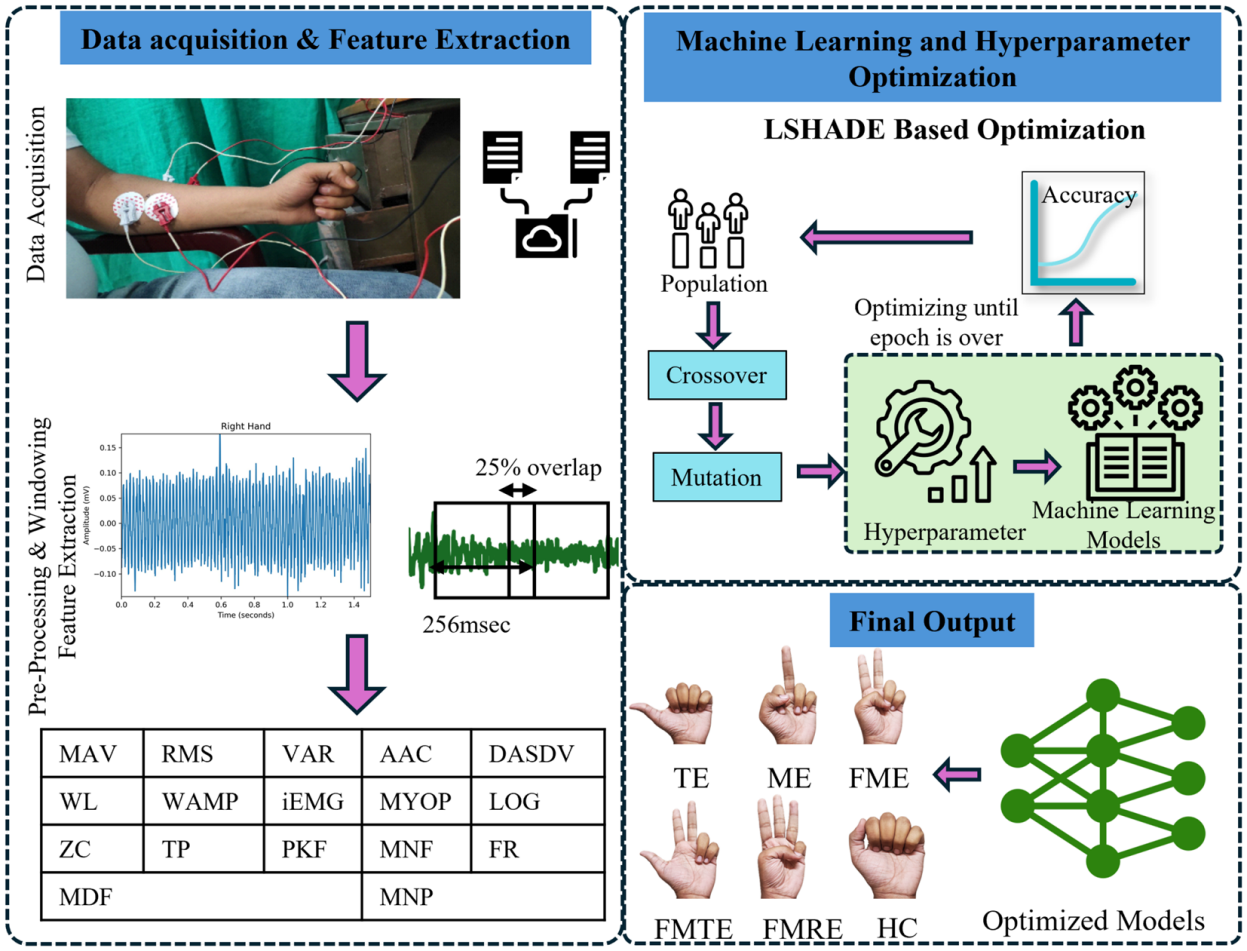
Proposed methodology of hand gesture identification. TE: Thumb Extension, ME: Middle Extension, FME: Fore + Middle Extension, FMTE: Fore + Middle + Thumb Extension, FMRE: Fore + Middle + Ring Extension, HC: Hand Close.

The following sections comprise this study; a brief introduction is presented in section. In section, the procedure of dataset preparation and feature extraction is presented. Section is divided into ten subsections discussing the machine learning models. Section is divided into ten subsections discussing the optimization technique that is used during the fine-tuning of hyperparameters of machine learning models. Results have been discussed in section . The study is concluded in section with the future scope.

## Methodology

The proposed methodology for classifying hand gesture movements is shown in Fig. 1. It demonstrates the classification stages, including dataset preparation, hyperparameter optimization, and classification using machine learning models. Initially, the forearm muscles’ sEMG data are collected and preprocessed. Relevant features are extracted following data preprocessing and then applied to machine learning models to classify the six hand gesture activities. A high degree of accuracy is most important in the field of medical for mapping assistive devices to tasks similar to natural ones. The performance of machine learning models is highly dependent on their hyperparameters because they control the learning process. To enhance the classification performance, ten distinct optimization algorithms are considered to optimize the parameters of machine learning algorithms. The subsections of the proposed methodology are explained as follows:

### Dataset preparation

In this study, data for hand gesture identification are acquired from a group of four willing participants. Prior to their involvement, each participant provided written informed consent, demonstrating their understanding and willingness to take part in the research. The anthropometric data of the participants who participated in the data acquisition process are outlined in Table 1.

**Table 1:** Anthropometric information about participants: Gender (F: Female M: Male), Age, Weight, Height, Forearm length (Forearm length: The measurement from the inner side of the elbow to the wrist line), Forearm circumference (The circumference of the forearm next to the rubber ring).

Participant	Gender	Age	Weight (Kg)	Height (cm)	Forearm Length (cm)	Forearm Circumference (cm)
M1	M	28	79	170	27.5	26
M2	M	24	81	178	27.5	27
F1	F	22	67	165	27	21
F2	F	26	62	163	28	22

**Fig. 2:**
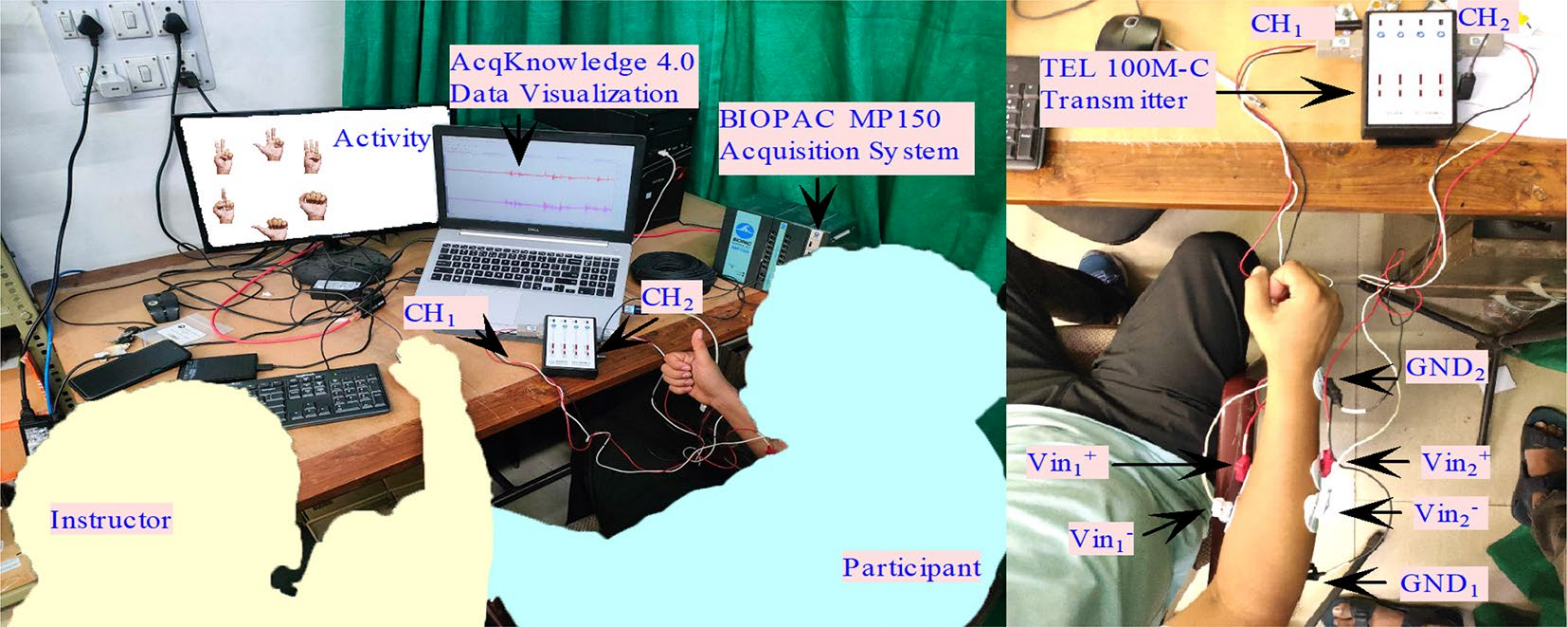
Hardware set up for the sEMG data acquisition.

The sEMG signals are collected using the AcqKnowledge software version 4.4, which is integrated with the BIOPAC MP150 system, which is well-known for its expertise in EMG data gathering and signal processing. Figure 2 depicts the configuration utilized throughout the data acquisition process. It shows the hardware instrumentation for data acquisition by placing disposable Ag/Ag-Cl surface electrodes on the participant’s forearm muscle to measure the sEMG signal. The extensor digitorum and flexor pollicis longus muscles are specifically targeted for signal assessment^40,41^. Based on anatomical landmarks, surface electrodes were placed on the forearm at one-third of the distance between the elbow and the wrist to ensure standardized placement across all subjects^42–44^. The sEMG signals are captured with a shielded cable electrode coupled to a TEL-100MC filter amplifier set to 1000 gain. This setup provided signal amplification and noise reduction. The data are collected at a sampling rate of 2 KHz to provide high-frequency resolution and a precise representation of muscle activation during hand gesture activities.

Four healthy adult male and female participants signed up for this study. The participants are asked to perform activities in a pre-listed sequence. A total of six activities are considered. Each of these activities is carried out in three stages, and during the task phase, the participant is instructed to meditate and relax.

**Step 1:** Pre-Task Stage: At this stage, the participants are briefed about the activity two minutes prior to performing the actual task.

**Step 2:** Task Stage: At this stage, the participants are asked to continuously perform the task for an interval of 40 seconds.

**Step 3:** Post-Task Stage: After the completion of each activity, the participants are given time to rest. Muscle signals can deteriorate due to fatigue in the muscles.

### Preprocessing

The collection of sEMG signals from the muscle is caused due to the action of muscle fibers at the time of muscle contraction. It is affected by unwanted external influences in the form of noise, such as signal line electromagnetic noise, electrode noise, and broadband noise^45^. Therefore, it is quite essential to reduce the noise of the original signal to provide accurate and effective data for feature extraction. Low frequencies of 1-10 Hz, which do not contain important information and are contaminated by movement artifacts, should often be rejected. Power source radiation, often known as Power-Line Interference, is a 50 Hz ambient noise. The influence of this noise can be reduced by using a narrow-band notch filter. Overall, these steps, which serve to reduce noise and prepare the EMG signal estimate, are used in this study:

1. Passing the 50 Hz notch filter on the narrowband signal. The notch filter’s transfer function is:
  1 $$\begin{aligned} W(s) = M\frac{s^4+s^2b_{2}+b_0}{s^4+s^3a_3+s^2a_2+sa_1+a_0} \end{aligned}$$
  Where $b_n$ and $a_n$ are the transfer function coefficients, *M* is the scale transmission coefficient of the filter^46^.
2. A $4^{th}$ order Butterworth band-pass filter having a cutoff frequency between 10 and 500 Hz. The band-pass filter’s transfer function is:
  2 $$\begin{aligned} W(s) = H\frac{s^4}{s^4+a_3s^3+a_2s^2+a_1s+a_0} \end{aligned}$$
  *H* is an arbitrary multiplicative constant, $a_n$ are the transfer function coefficients^47^.

**Fig. 3:**
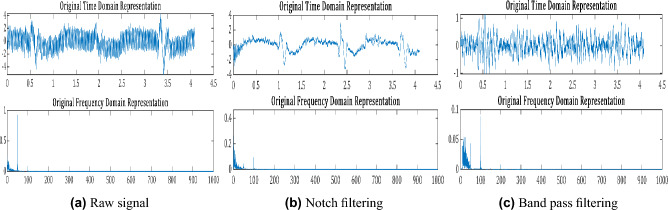
EMG preprocessing example for participant M1.

Figure 3 is present as an example of the sEMG signal, so that the impacts of each preprocessing step may be clearly observed. While the figure shows that the raw sEMG signals are noisy, the extreme frequency components are removed following a two-stage filtering process. The preprocessed signal (i.e., Fig. 3) will be used to extract features for machine learning models in the following section.

### Feature extraction

**Fig. 4:**
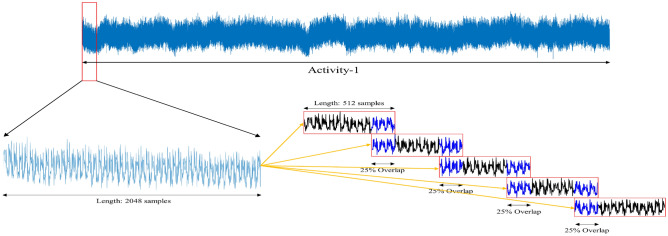
Overlapping windowing technique is used to segment the signal into windows of 256ms. Features are extracted from these windows of the signal rather than the entire signal.

The practical units of data from raw signals are known as features. Selecting an appropriate feature is one of the most important factors of classification accuracy. The process of turning raw data into input that the classification system can use is known as feature extraction. The extraction of features from the signal has segmented the signal into intervals of 256 msec windows with an overlapping length equivalent to 25% of the signal^48^. The windowing approach is utilized in this case because the entire data set is too large to analyze and includes redundancy, and the instantaneous EMG signal sample has insufficient information on overall muscle activity. Figure 4 represents windowing with the overlapping segmentation of signals.

To reduce the possibility of reporting unsatisfactory performance in the dataset, extract the most beneficial features for sEMG data based on various characteristics and outcomes. These signal features can be obtained using time domain analysis, frequency domain analysis, or both. In this study, 17 such features have been extracted from each signal segment. Among the features, eleven are extracted in the time domain and six in the frequency domain. The mathematical formulation of features is shown in Table 2.

**Table 2:** Features of the time-frequency domain.

SN	Feature Name	Mathematical expression	SN	Feature Name	Mathematical expression
Time Domain Features					
1	Mean Absolute Value (MAV)	$$ \frac{1}{N} \sum _{p=1}^{k}\left| x_{p} \right| \ $$	2	Root Mean Square (RMS)	$$ \sqrt{\frac{1}{N}\sum _{p=1}^{k}\left| x_{p} \right| ^{2}}\ $$
3	Variance (VAR)	$$ \frac{1}{N-1}\sum _{i=1}^{p}x_{p}^{2}\ $$	4	Average Amplitude Change (AAC)	$$ \frac{1}{N}\sum _{p=1}^{N-1}\left| x_{p+1}-x_{p} \right| \ $$
5	Difference Absolute Standard Deviation Value (DASDV)	$$ \sqrt{\frac{1}{N-1}\sum _{p=1}^{N-1}(x_{p+1}-x_{p})^{2}}\ $$	6	Zero Crossing (ZC)	$$\begin{aligned} \sum _{p=1}^{N-1}f(x_{p})\\ where \;f(x_{p})= {\left\{ \begin{array}{ll} 1 & \text {if } ({x_{p}> 0} \; and\; x_{p+1}< 0)\;\\ & or\; ({x_{p}< 0} \; and\; x_{p+1} > 0) \\ 0, & \text {otherwise} \end{array}\right. } \end{aligned}$$
7	Waveform Length (WL)	$\frac{1}{N-1}\sum _{p=1}^{N}\left| x_{p}\right| ^2$	8	Willson Amplitude (WAMP)	$$\begin{aligned} \sum _{p=1}^{N-1}f(\left| x_{p+1}-x_{p} \right| )\\ where \;f(x_{p})= {\left\{ \begin{array}{ll} 1 & \text {if } {x\ge Threshold} \\ 0, & \text {otherwise} \end{array}\right. } \end{aligned}$$
9	Integrated Electromyogram (iEMG)	$\sum _{p=1}^{N}\left| x_{p}\right|$	10	Myopulse (MYOP)	$$\begin{aligned} \frac{1}{N}\sum _{p=1}^{N}f( x_{p})\\ where \; f(x_{p})= {\left\{ \begin{array}{ll} 1 & \text {if } {x\ge Threshold} \\ 0, & \text {otherwise} \end{array}\right. } \end{aligned}$$
11	Log Detector (Log)	$e^{\frac{1}{N}\sum _{p=1}^{N} log(\left| x_p \right| )}$			
Frequency Domain Features					
12	Total Power (TP)	$\sum _{k=1}^{M}P_{k}$	13	Peak Frequency (PKF)	$\frac{1}{2}\sum _{i=1}^{M}P_{k}$
14	Frequency Ratio (FR)	$\frac{\sum _{k-LLC}^{ULC}P_{k}}{\sum _{LHC}^{UHC}P_{k}}$	15	Mean Frequency (MNF)	$\frac{\sum _{k=1}^{M}f_{k}P_{k}}{\sum _{k=1}^{M}P_{k}}$
16	Median Frequency (MDF)	$\frac{1}{2}\sum _{k=1}^{M}P_{k}$	17	Mean Power (MNP)	$\sum _{k=1}^{M}\frac{P_{k}}{M}$

Here $x_p$ is the $p^{th}$ input sample of sEMG signal; N is the total no. of samples, $P_k$ is the power at $k^{th}$ frequency, and M is the length of the power spectrum density^49^

## Machine learning classifiers

This study uses ten machine learning models to classify hand gestures. For the employed models, performance parameters are evaluated, and the best classifier is selected to help in the improvement of gesture recognition. For the recognition of hand gestures, machine learning models are as follows: Decision Tree(DT), Random Forest (RF), Adaboost (ADB), Bagging (BAG), Gradient Boosting (GB), Support Vector Machine (SVM), Logistic Regression (LR), Naive Bayes (NB), k-Nearest Neighbour (KNN)^49–51^, and Extra Tree (ET). The brief description of the most accurate machine learning model, ET is as follows:

### Extra Tree (ET)

Extra Tree refers to extremely randomized decision trees. This ensemble has a similar number of decision trees as the random forest but differs in how randomization is incorporated during training. Training and splitting of branches are the primary aspects that set this algorithm apart from the random forest. The ET algortihm uses the classical top-down approach to construct an ensemble of the unpruned decision or regression trees. Its two primary distinctions from previous tree-based ensemble approaches are that it splits nodes by selecting cut-points randomly and grows the trees using the entire learning sample (rather than a bootstrap replica).

The method mainly comprises of two parameters: no. of attributes *K* randomly selected at each node and the minimal sample size $n_{min}$ for splitting a node. Here, attribute refers to a specific input variable used in an ET. The number of trees of the ensemble parameters *M*, *K*, and $n_{min}$ have different effects: *K* determines the strength of the attribute selection process, $n_{min}$ the strength of averaging output noise, and *M* the strength of the variance reduction of the ensemble model aggregation. These parameters might be automatically or manually altered to the particulars of the problem. The information gain is specifically normalized in the measured score for classification. This measure can be obtained for a sample *S* and a split *s*:

3 $$\begin{aligned} S_c(s,S)=\frac{2I_c^s(S)}{H_s(S)+H_c(s)} \end{aligned}$$

Where $H_c(s)$ is the log entropy for the classification, $H_s(S)$ is the split entropy, and $I_c^s(S)$ is the mutual information of the split outcome and the classification. The algorithm’s remarkable efficiency directly results from these properties^52,53^.

## Optimization techniques

Optimization techniques are generally applied for hyperparameter tuning of machine learning models and thus help in improving their performance. These are helpful in finding the optimal parameters for machine learning models. In this study, ten different optimization algorithms have been employed to improve the performance of the extra tree (ET) machine learning model. Moreover, these algorithms have been compared, and the best algorithm among them has been evaluated. Ten different optimization algorithms that have been employed to improve the accuracy of ET are Particle Swarm Optimization (PSO), Atom Search Optimization (ASO), Grey Wolf Optimization (GWO), Genetic Algorithms (GA), Artificial Rabbits Optimization (ARO), Biogeography-Based Optimization (BBO), Artificial Ecosystem-based Optimization (AEO), Harmony Search (HS), Water Cycle Algorithm (WCA)^54–56^, and Linear Population Size Reduction Success-History Adaptation Differential Evolution (L-SHADE). A brief introduction to the highly dominating L-SHADE algorithm has been mentioned in the following subsections:

### Linear population size reduction success-history adaptation differential evolution (L-SHADE)

L-SHADE is one of the state-of-the-art DE algorithms, an adaptive DE (differential evolution) that incorporates the linear success history-based parameter adaption. DE is a more straightforward method that uses a lesser number of parameters. It has been enhanced using the Linear Population Size Reduction is known as LSHADE^57^.

In this algorithm, population is represented as $x_i = (x_1,\ldots , x_N)$, $i = 1,\ldots , n,$, where, *N* is the dimension of the target and *n* is the population size. It uses Crossover rate *CR*, scaling factor *SF*, and a memory set of historical memory cells *H* containing values $M_{SF, K}, M_{CR, K}$ to generate new parameters for the crossover and mutation in every iteration^58^. These parameters are sampled using randomly chosen memory index $k \;\epsilon \; [1, H]$ as follows:

4 $$\begin{aligned} SF= & randc(M_{SF, K},0.1) \end{aligned}$$

5 $$\begin{aligned} CR= & randn(M_{CR, K},0.1) \end{aligned}$$

where *randc*(*a*, *b*) is a random value generated by the Cauchy distribution, and *randn*(*a*, *b*) is a randomly generated value by the normal distribution with position and scale parameters (*a*, *b*).

The crossover rate and scaling factor value are in the range [0, 1]. The crossover and scaling factor improvement is stored in the array $S_{SF}$, $S_{CR}$, together with the fitness difference stored in $\Delta f$. The updation in the memory cell with index *h* increasing from 1 to *H* with every generation is as follows:

6 $$\begin{aligned} mean_{wl}=\frac{\sum _{j=1}^{\left| S \right| }w_{j}S_{j}^{2}}{\sum _{j=1}^{\left| S \right| }w_{j}S_{j}} \end{aligned}$$

where, $w_j=\frac{\Delta f_{i}}{\sum _{k=1}^{\left| S \right| }\Delta f_{k}}$ and $\Delta f_{j}=\left| f(u_j) -f(x_j)\right|$ and *S* from the $S_{SF}$ or $S_{CR}$. The previous parameter values are used to set the new ones with updated parameters *c* as follows:

7 $$\begin{aligned} M_{SF, k}^{g+1}= & c.M_{SF,k}^{g}+(1-c)mean_{wl}(SF) \end{aligned}$$

8 $$\begin{aligned} M_{CR, k}^{g+1}= & c.M_{CR,k}^{g}+(1-c)mean_{wl}(CR) \end{aligned}$$

where *g* is the current generation number. In general, the updated parameter *c* is set at 0.5.

In the L-SHADE algorithm, the number of individuals decreases linearly as it uses linear population size reduction. Therefore, at the end of each iteration, the size of NP is recalculated, and the worst individuals are eliminated from the population. The population size is updated as follows:

9 $$\begin{aligned} NP_{g+1} = round\left( \frac{{NP_{min}}-{NP_{max}}}{NF_{max}}NF + NP_{max} \right) \end{aligned}$$

where, $NP_{min}$, $NP_{max}$ are the minimum and maximum population sizes. Similarly, *NF*, $NF_{max}$ are the current and maximum number of function evaluations, respectively.

## Experimental results and analysis

This section explains the experimental results and their analysis of acquired hand gesture data classification based on the sEMG signal. On the acquired dataset, ten machine learning algorithms have been applied. Initially, the data is split into two parts without disturbing the original sequence. The first part contained 70% of the data from the beginning, which is used during training, and the remaining 30% of the data is reserved for testing. The original sequence is not disturbed to keep the temporal order intact. Four cases of different participants (M1, M2, F1, F2) are considered to generalize the study on classifiers. The four performance indices of these classifiers are recorded as shown in Table 3. From the table, it can be concluded that the ET classifier gives the highest accuracy of around 85.22%, 87.37%, 87.37%, and 76.61% in the case of participants M1, M2, F1, and F2, respectively, as compared to other studied classifiers. The precision corresponding to the same classifier is 86.64%, 87.85%, 87.89%, and 76.72% in the case of participants M1, M2, F1, and F2, respectively. The recall value associated with the ET classifier is 85.21%, 87.37%, 87.37%, and 76.61% in the case of participants M1, M2, F1, and F2, respectively. And the F1-Score is 85.27%, 87.15%, 86.99% and 76.44% in case of participant M11, and F2, respectively. Next to ET, BAG has the maximum accuracy in the case of participant M1, while in the case of participants M2, F1, and F2, LR, GB, and RF have the second-best accuracy, respectively.

**Table 3:** Performance analysis of machine learning classifiers (in %).

Participant	Performance Measures	ADB	BAG	DT	GB	KNN	LR	NB	RF	SVM	ET
M1	Accuracy	70.16	84.14	79.84	83.87	70.43	75.27	64.52	82.80	67.20	**85.22**
	Precision	70.88	85.71	82.42	85.85	72.75	77.19	68.07	84.42	71.87	86.64
	Recall	70.16	84.14	79.84	83.87	70.43	75.27	64.52	82.80	67.20	85.21
	F1-Score	69.21	84.15	80.10	84.24	69.42	75.68	64.36	82.90	65.88	85.27
M2	Accuracy	49.73	78.23	72.58	83.06	78.49	83.87	59.95	83.06	69.89	**87.37**
	Precision	67.86	79.16	74.83	84.29	79.26	84.77	65.46	84.24	74.77	87.85
	Recall	61.29	78.23	72.58	83.06	78.49	83.87	59.95	83.06	69.89	87.37
	F1-Score	61.55	77.59	71.66	82.62	78.30	84.07	58.77	82.82	68.98	87.15
F1	Accuracy	49.73	86.29	86.29	86.83	85.75	86.83	84.14	86.02	69.89	**87.37**
	Precision	30.53	86.39	86.38	87.50	85.90	87.77	84.14	86.60	68.69	87.89
	Recall	49.73	86.29	86.29	86.83	85.75	86.83	84.14	86.02	69.89	87.37
	F1-Score	35.94	86.18	86.20	86.33	85.57	86.20	84.14	85.38	66.30	86.99
F2	Accuracy	56.45	74.46	68.01	75.00	56.99	71.77	61.02	75.27	64.52	**76.61**
	Precision	59.42	74.99	68.44	75.34	57.72	72.43	62.36	75.60	64.76	76.72
	Recall	56.45	74.46	68.01	75.00	56.99	71.77	61.02	75.27	64.52	76.61
	F1-Score	55.14	74.27	68.17	74.77	56.32	71.86	61.15	75.00	64.24	76.44

**Table 4:** Optimized value of hyperparameter.

S.No.	Hyperparmeter	Range	Optimal Value			
			M1	M2	F1	F2
1	No. of Estimators	$\left[ 10,100 \right]$	20	49	11	31
2	Criterion	$\left\{ gini,entropy \right\}$	gini	gini	gini	gini
3	Minimum samples split	$\left[ 2,30 \right]$	4	2	3	17
4	Maximum Features	$\left[ 2,34 \right]$	34	11	2	11
5	Maximum Depth	$\left[ 1,25 \right]$	14	18	16	14

Additionally, it can be observed that GB appears at least once among the top three classifiers in all the cases. While LR appears as among the best three classifiers in the case of participants M1, M2, and F1 only. The above-discussed machine learning models have learning hyperparameters with predetermined values. These values of hyperparameters for a classifier with higher accuracy are optimized. Optimization technique with 500 iterations for 30 runs to get a highly accurate model produced by the optimization algorithm to obtain the optimal value of the hyperparameter.

Considerable hyperparameters for the optimal value are provided in Table 4 while tuning the machine learning model. Five tuneable hyper-parameters of the ET algorithm are selected for this purpose.

**Table 5:** Accuracy (in %) comparison of the hybrid of Extra Tree classifier with optimization techniques.

Model	M1	M2	F1	F2	Mean
ET	85.22	87.37	87.37	76.61	84.14
ET+AEO	88.17	89.78	90.86	79.83	87.16
ET+ARO	88.44	90.32	90.59	80.10	87.36
ET+ASO	87.90	89.24	89.51	79.56	86.55
ET+BBO	87.36	89.78	89.51	79.03	86.42
ET+GA	88.17	90.32	90.59	79.83	87.23
ET+GWO	88.44	90.32	90.32	80.10	87.30
ET+HS	87.09	87.90	87.36	77.95	85.08
ET+PSO	88.44	89.78	90.86	80.37	87.36
ET+WCA	88.44	90.05	90.32	79.83	87.16
ET+L-SHADE	**88.97**	**90.59**	**91.12**	**80.91**	**87.89**

The first hyperparameter of the extra tree classifier is the number of estimators, which can range from 10 to 100. The number of estimators decides the number of decision trees. The second hyperparameter is the classification criterion. It can be either *Gini* or *Entropy*.

**Fig. 5:**
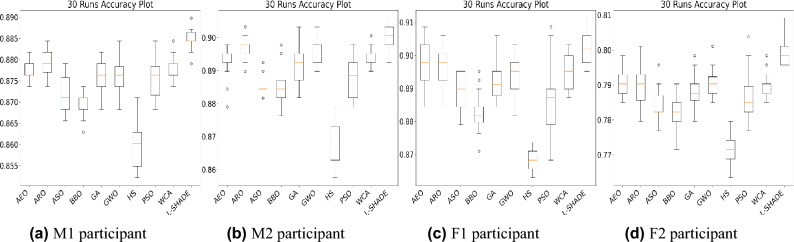
Accuracy plot of optimization technique for hyperparameter tuning.

The criteria govern the splits in a decision tree. The third hyperparameter is the minimum sample split. Its range is set from 2 to 30; it decides the minimum number of splits of a node. The fourth hyperparameter is the maximum number of features for the selection, which ranges from 2 to 34. The fifth hyperparameter is the model’s maximum depth, which ranges from 1 to 25. It decides the level of tree growth. The hyperparameters are tuned separately for each participant, and the optimal ones are listed in Table 4. Most notably from the table, the *Gini* criterion appears consistently as the optimal classification criterion parameter in ET. At the same time, the other parameters vary across participants.

**Table 6:** Performance analysis of best classifier after optimizing the hyperparameter.

Participant	Performance parameter	ET	ET + L-SHADE
M1	Accuracy	85.22	**88.97**
	Precision	85.24	89.89
	Recall	83.87	88.98
	F1-Score	83.92	89.05
	Time (ms)	9.00	2.91
M2	Accuracy	87.37	**90.59**
	Precision	87.85	91.02
	Recall	87.37	90.59
	F1-Score	87.15	90.59
	Time (ms)	7.50	4.08
F1	Accuracy	87.37	**91.12**
	Precision	87.89	91.30
	Recall	87.37	91.13
	F1-Score	86.99	91.08
	Time (ms)	7.00	2.67
F2	Accuracy	76.61	**80.91**
	Precision	76.72	81.15
	Recall	76.61	80.91
	F1-Score	76.44	80.61
	Time (ms)	10.98	3.00

Figure 5 shows four boxplots corresponding to four different cases of participants. The boxplot is used to indicate variation in the accuracy of the ET classifier caused by the ten optimizers over the 30 runs. It is observed that classification accuracy remains nearly consistent when L-SHADE is used as an optimizer to tune the hyperparameters of the ET classifier. Also, the consistent value is near to the maximum value of accuracy, which justifies the applicability of the L-SHADE optimizer, as compared to the other optimizers, in this study.

Table 5 represents the improved accuracy obtained after optimally tuning the parameters. Accuracy improvement as seen in the case of ET with L-SHADE is maximum, which is about 88.97%, 90.59%, 91.12%, and 80.91% in the case of participants M1, M2, F1, and F2. The average improvement is about 4% which can provide great relief to users of the prosthetic arm while doing daily chores. Another such application also reduces the chances of mishaps caused during teleoperation. Both of which remain the end effect of this study.

After hyperparameter tuning, the model’s performance measure with and without the optimization is recorded in Table 6. And it can be observed that performance measure accuracy, precision, recall, and F1 score improve with optimization, as well as time is reduced for the participants M1, M2, F1, and F2 when the hyperparameter is optimized.

**Fig. 6:**
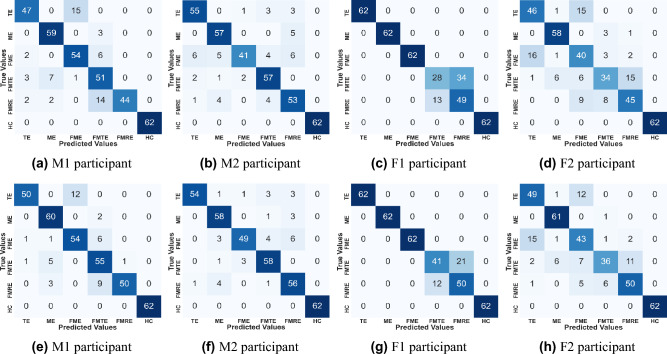
Confusion matrix of classifier before and after the hyperparameter optimization.

A confusion matrix illustrates the performance of a classification technique. It contains information about the actual and expected labels that a model evaluates. In the confusion matrix, the diagonal element represents the data point that has been correctly categorized. Figure 6 presents the confusion matrix of the Extra Tree classifier, in which Fig. 6(a)-(d) represents the confusion matrix corresponding to the participants before hyperparameter tuning, while the rest of the subfigures (Fig. 6(e)-(h)) represent the confusion matrix corresponding to the participants after hyperparameter tuning. Figure 6(a) can be interpreted as 44 samples of TE, 59 samples of ME, 53 samples of FME, 47 samples of FMTE, 47 samples of FMRE, and 62 samples of HC are correctly classified, whereas 18 samples of TE, 3 samples of ME, 9 samples of FME, 15 samples of FMTE, 11 samples of FMRE, and zero samples of HC are incorrectly classified. Similarly, observations can be drawn from the rest of the confusion matrices. However, it can be visually compared that the number of correct classifications improves for at least two activities in all cases after tuning.

### Assessment of model generalizability

**Fig. 7:**
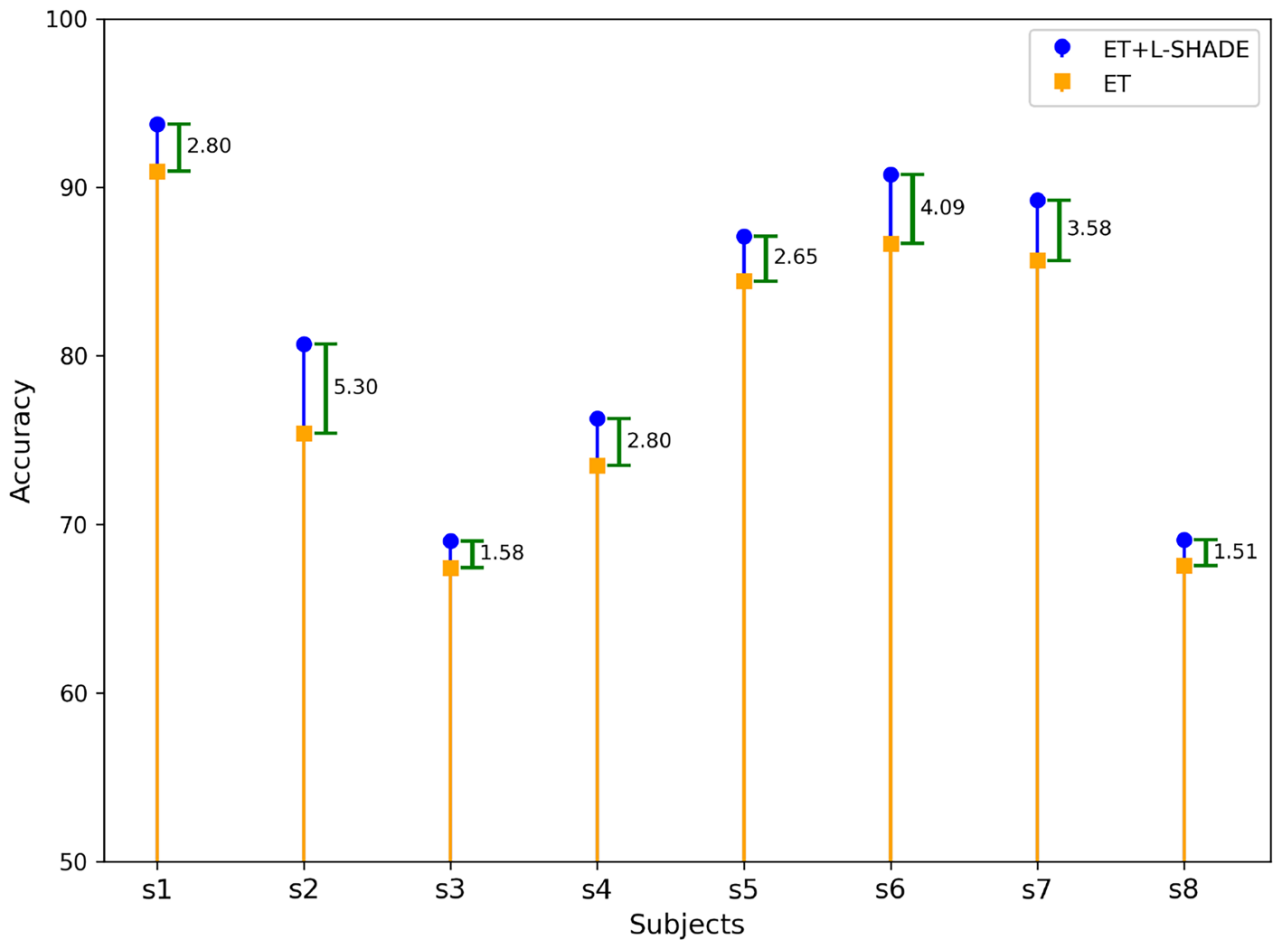
Accuracy analysis of the public dataset.

The proposed framework’s generalizability is assessed with the help of validation on the publicly available, more diverse gesture data, and also through the statistical analysis of the model performance through statistical hypothesis testing methods, along with checking the practical viability based on the cross-session performance. The following subsection illustrates the evaluation with a public dataset and the statistical analysis as well on the cross-session viability of the proposed framework.

#### Evaluation with publicly dataset

The generalizability of the proposed framework, optimized ET with the L-SHADE, was evaluated using the public data repository from R. N. Khushaba et al.^59^, which includes datasets of 15 different classes of hand gestures. During robustness analysis, the same steps are followed, and a similar environment is set up. After ensuring uniformity in the environment, the datasets are analyzed using the two best machine learning models: our proposed model, ET+L-SHADE, and the second most accurate model on the collected data, ET.

From these analyses, it was observed that the proposed architecture achieved the highest accuracy among all subjects (a total of eight). The accuracy attained by the subjects is shown in Fig. 7. The figure clearly shows that the accuracy achieved by subjects s1-s8 with the proposed ET+L-SHADE model had a mean increase in accuracy of approximately 3.0% for all subjects. Thus, in this setup, ET+L-SHADE attained the highest accuracy for both the collected and the publicly available datasets.

#### Evaluation based on statistical analysis

**Table 7:** T-test statistics for data comparison.

Data	t-statistic	p-value
Acquired	17.03	0.0004
Public	6.78	0.0003

To validate the significance of the proposed framework, L-SHADE-based optimized Extra Tree MLC results achieved high accuracy. For the statistical significance check, a t-test was conducted on both datasets, acquired. The result of the t-test with the statistic parameter t-value and p-value is illustrated in Table 7. The results on both datasets signify that the p-value is less than 0.05. It means the proposed approach has a significant improvement over the base classifier, and the proposed approach consistently outperforms among both datasets.

#### Cross-session evaluation

**Table 8:** Cross-session classification accuracy using ET and ET+L-SHADE (%).

Subject	Training Session	Testing Session	ET	ET+L-SHADE
s1	S1	S2	83.50	86.80
	S1	S3	81.78	82.03
	S2	S3	86.22	86.92
s2	S1	S2	79.22	79.74
	S1	S3	60.95	63.05
	S2	S3	76.44	79.75
s3	S1	S2	63.17	65.89
	S1	S3	45.33	46.35
	S2	S3	70.03	70.67
s4	S1	S2	61.04	64.14
	S1	S3	55.62	56.95
	S2	S3	72.13	73.52
s5	S1	S2	86.99	88.99
	S1	S3	67.56	68.57
	S2	S3	76.70	79.94
s6	S1	S2	71.33	72.94
	S1	S3	63.87	66.16
	S2	S3	85.52	86.40
s7	S1	S2	77.93	78.09
	S1	S3	69.78	71.68
	S2	S3	84.51	85.84
s8	S1	S2	74.05	74.37
	S1	S3	60.76	61.02
	S2	S3	67.62	68.30

To assess the generalizability of the proposed framework, cross-session variability was checked on the publicly accessible dataset. The dataset comprises recordings from three trial sessions (S1, S2, S3) throughout the acquisition. For the generalizability check, this study assessed that one session was designated for training, while the remaining two sessions were used for testing. This process was repeated for each subject to establish. Such an arrangement replicates a real-world scenario where factors like posture, muscle fatigue, and the placement of the electrode cause disturbances in the signals over time.

The significant results of the cross-session are illustrated in Table 8. The table summarizes the eight subjects’ data performance with respect to the proposed (ET+L-SHADE) in comparison to the ET across various session combinations for individual subjects. The results show outperformance among all the subjects with every session; ET+L-SHADE beats the base classifier ET. Notably, subjects s1, s2, and s5 show gains that are more noticeable in every cross-session compared to the others. These findings highlight the enhanced adaptability and generalizability of the proposed model.

Following the discussion on model generalizability, we elaborate on the computational efficiency to further support the practical applicability of the proposed method. The computational complexity of the L-SHADE-optimized Extra Trees framework is approximately ${\mathcal {O}}(N.P.T.n.log n.d)$, where *N* represents the no. of generations, *P* denotes the size of the population, *T* is the no. of trees, *n* signifies the number of training samples, and *d* is the depth of the tree.

However, once the model is trained, the inference complexity is reduced to ${\mathcal {O}}(T.d)$ per data sample. This low inference cost makes the proposed framework highly suitable for real-time applications. Furthermore, the Extra Trees classifier supports easy pruning and delivers low-latency decision-making, reinforcing the framework’s practical applicability in real-time prosthetic technologies.

### Comparison with state-of-the-art studies

This study introduces an L-SHADE-optimized learning framework for sEMG-based hand gesture recognition (HGR). The superior performance of the proposed framework has been validated using both a self-acquired dataset and a publicly available dataset. Existing literature primarily focuses on improving gesture recognition accuracy, often without optimizing the hyperparameters of machine learning classifiers. This critical aspect is frequently overlooked by researchers. Due to the fact that they are trying to develop a new classifier in place of tuning the parameters of the classifier. To address this gap, we propose an L-SHADE-optimized Extra Tree classifier for enhanced hand gesture recognition. Along with a comparative analysis, a presentation of the ten optimization algorithms that enhance the performance of the subject-specific classifier is provided.

**Table 9:** Detailed comparison with existing studies.

Ref.	# Ch.	# Gst.	# Feat.	# Classifiers	# OPT	Validated		Accuracy (%)
						Acq.	Pub.	
^60^	12	17	4	4(3M+1D)	-	✗	✓	85.9
^61^	4	10	-	3D	1	✗	✓	88.71
^62^	6	10	-	1D	2	✓	✗	92
^63^	6	16	5	4D	-	✓	✗	91.25
^64^	-	27	9	4(3D+1M)	1	✗	✓	85
^65^	6	7	10	4M	-	✓	✗	97.18
^66^	8	11	-	4M	-	✓	✓	84.85, 81.93
This study	2	6	17	10M	10	✓	✓	Mean: 87.89, 82.00

Ch. = Channel, Gst. = Gestures, Feat. = Features Ref. = Reference, # = Number of, OPT = Optimization Techniques,

Acq. = Acquired Dataset, Pub. = Public Dataset, M = Machine Learning, D = Deep Learning

Table 9 summarizes state-of-the-art studies that utilize hyperparameter tuning of machine learning classifiers (MLCs), highlighting their best performances for comparison with our study in terms of key parameters such as no. of channels, no. of classifiers, no. of used optimization techniques, and data for validation. According to the literature, while model performance has generally improved, the aspect of systematic hyperparameter tuning using diverse optimization techniques remains largely underexplored. Therefore, in this study, a detailed comparison is provided with two such studies on the low-end processors and fewer storage units for hardware implementation of a machine learning classifier, which makes the device economical and lighter. One more important thing these machine learning classifiers are easy to interpret and make trustworthy.

## Conclusion and future scope

This study introduced a framework for the optimal way to identify hand gestures using fine-tuned learning models. It helps in the smooth functioning of the prosthetic or assistive devices at the user’s end without many glitches in operation. This is achieved in the study following a directed procedure in which ten machine learning models are initially tested for suitability around the 34 considered features for identifying gestures. Among the ten classifiers used, ET is the best in terms of the performance indices compared to other studied classifiers, with a mean accuracy of 84.14%. The hyperparameters of the ET classifiers are further exploited, and it is observed that ET has five tunable hyperparameters. These five tunable hyperparameters are further tuned using ten different optimization algorithms to improve the accuracy further. It is observed that the accuracy of the ET classifier after hyperparameter tuning with the L-SHADE optimization algorithm improves by about 4%, which is comparable to better than other optimizers. The mean accuracy of the classification of six hand gestures performed by the four participants using the hybrid approach of the Extra Tree classifier and L-SHADE optimization algorithm is 87.89%. Additionally, the generalizability of the ET+L-SHADE framework was evaluated on a publicly available dataset. The results demonstrate that the mean accuracy improvement exceeds 3%, further validating the effectiveness of the proposed approach. So, the proposed technique can be used in future work to recognize more hand gestures in a real-time application of controlling the prosthetic limb. Future research will also address the study’s shortcomings by investigating optimization methods beyond those examined and looking at the integration of other deep learning and machine learning models for parameter adjustment. Additionally, future work will involve evaluating the proposed framework on amputee subjects to better assess its practical applicability and performance in real-world prosthetic control scenarios. In these scenarios, the electrode placement variation may introduce challenges that affect signal quality and system performance. Addressing these limitations may enhance the generalizability, robustness, and reliability. Future research will focus on developing standardized electrode placement protocols and adaptive signal processing techniques to mitigate the impact of such variability of the electrode in real-world applications. Also, deep learning models such as transfer learning and incremental learning are still intriguing for further research in resource-rich environments; they have not been used because of their high computational complexity, high hardware implementation costs, and less interpretable aspects that are crucial in real-time and wearable prosthetic applications.

## Data Availability

The datasets used and/or analysed during the current study are available from the corresponding author on reasonable request.
